# Child Fitness and Father’s BMI Are Important Factors in Childhood Obesity: A School Based Cross-Sectional Study

**DOI:** 10.1371/journal.pone.0036597

**Published:** 2012-05-31

**Authors:** Sinead Brophy, Anwen Rees, Gareth Knox, Julien Baker, Non E. Thomas

**Affiliations:** 1 College of Medicine, Swansea University, Swansea, Wales, United Kingdom; 2 School of Sport, University of Wales Institute, Cardiff, Wales, United Kingdom; 3 University of West Scotland, Paisley, Scotland, United Kingdom; 4 School of Human and Health Sciences, Centre for Children and Young People’s Health and Well-Being, Swansea University, Swansea, Wales, United Kingdom; Postgraduate Medical Institute & Hull York Medical School, University of Hull, United Kingdom

## Abstract

**Background:**

This study examines obesity and factors associated with obesity in children aged 11–13 years in the UK.

**Methods:**

1147 children from ten secondary schools participated in a health survey that included blood samples, fitness test and anthropometric measures. Factors associated with obesity were examined using multilevel logistic regression.

**Findings:**

Of the children examined (490 male; 657 female) a third were overweight, 1 in 6 had elevated blood pressure, more than 1 in 10 had high cholesterol, 58% consumed more fat than recommended, whilst 37% were classified as unfit. Children in deprived areas had a higher proportion of risk factors; for example, they had higher blood pressure (20% (deprived) compared to 11% (non-deprived), difference: 9.0% (95%CI: 4.7%–13.4%)). Obesity is associated with risk factors for heart disease and diabetes. Maintaining fitness is associated with a reduction in the risk factors for heart disease (high blood pressure and cholesterol) but not on risk factors for diabetes (insulin levels). In order of importance, the main risk factors for childhood obesity are being unfit, having an obese father, and being large at birth.

**Conclusion:**

The high proportion of children with risk factors suggests future interventions need to focus on community and policy change to shift the population norm rather than targeting the behaviour of high risk individuals. Interventions need to focus on mothers’ lifestyle in pregnancy, fathers’ health, as well as promoting fitness among children. Obesity was not associated with deprivation. Therefore, strategies should be adopted in both deprived and non deprived areas.

## Introduction

The World Health Organisation predicts that by 2015 approximately 2.3 billion adults will be overweight and more than 700 million will be obese. In 2005, at least 20 million children under the age of 5 were overweight [1]. Obesity can lead to many health complications, including hypercholesterolemia and hypertension, which in turn can lead to chronic health consequences. Cardiovascular disease (CVD) and diabetes are two chronic diseases which are rapidly increasing globally. Even though the health consequences of obesity are most commonly seen during adulthood, the underlying factors of these diseases often originate during childhood.

Many studies, including the Muscatine Study [2], [3] and the Bogalusa Heart Study [4], have convincingly shown that overweight and obesity during adolescence is a determinant of a number of CVD risk factors in adulthood. The Bogulasa Heart Study is the most extensive study examining the tracking of obesity throughout life. Results showed that BMI during childhood was significantly related to BMI during adulthood and this was present as early as age 2–5 yrs old [5]. Several other studies have supported this evidence, including data from the Newcastle Thousand Family Study, which showed that at age 50, those participants that had been above the 90^th^ centile at age 9–13 yrs were between five and nine times more likely to be obese at age 50. This was in comparison to those who had been in the lowest centiles [6].

This study examines prevalence of obesity and cardiovascular risk factors such as cholesterol level, fasting insulin, fasting glucose, adiponectin, high blood pressure and looks at the factors associated with obesity in children at age 11–13. As deprivation is associated with higher cardiovascular mortality in adults [7], the prevalence figures in this study were stratified by deprivation in order to examine the effect of socio-economic deprivation on obesity and CVD risk factors in children. It is anticipated these findings can be used to inform the development of interventions aimed at improving health in children aged 11–18.

## Results

A total of 1147 (38%) children (490 male: 657 female) out of a potential 3029 participated in the study. Those who participated were comparable to non-participants in terms of cardiovascular fitness as measured by the 20 MSFT (class average level 5.8 compared to sample average level 6.1). Eleven schools were approached to participate in the study, with one school in a deprived area declining as they did not want to be involved in research. Therefore, 90% of schools approached participated in the research. Of the 1147 participating children, 918 (81%) undertook blood sampling; in terms of BMI, there was no difference between those giving blood samples (20.58 kg/m^2^ (stdev: 3.81)) and those who did not (20.38 kg/m^2^ (stdev: 4.24)).

### Prevalence of Risk Factors

The prevalence of risk factors for CVD and T2DM were compared for deprived versus non-deprived schools (see Table 1). In all schools surveyed, there was a high proportion of children with at least one risk factor. For example, at least 1 in 3 children were overweight, 1 in 6 had elevated blood pressure, and more than 1 in 10 had high cholesterol. Moreover, the majority of children consumed more fat than recommended (58%), and many children (37%) were classified as unfit. Children attending schools located in a deprived area had a higher proportion of risk factors compared to children in non-deprived areas. For example, the proportion of children with high blood pressure was two times higher in deprived schools (20%), compared to non-deprived schools (11%) (See Table 1); whilst the number with impaired fasting glucose in deprived schools was five times that reported in non-deprived. Children from deprived schools were more likely to consume a high fat diet, and more prone to be unfit. There were 8.6% (92/1072) of children who could be considered to have the metabolic syndrome (overweight/high waist measurement and high cholesterol and high blood pressure and/or high fasting glucose). Of these children, 62 (67%) were from a deprived area.

**Table 1:** Prevalence of risk factors for CVD and T2DM by deprivation area of school.

Demographic data	Deprived schools (n = 5)	Non- deprived schools (n = 5)	Difference (95%CI)
% with free school meal entitlement (by school)	22%, 28%, 41%, 44%, 62%	2%, 4%, 5%, 6%, 20%	
Number participating (number eligible)	30% (516/1712)	48% (631/1317)	
Female gender	58.7% (303/516)	43.9% Male (277 Male: 354 Female)	2.6% (95%CI: −3.1% to 8.3%)
Ethnic minority group	16.2% (51/315)	9.5% (33/348)	5.9% (95%CI: 3.6% to 8.5%)*
**Risk factors**			
% overweight	35.2% (177/503)	29.5% (175/594)	5.7% (95%CI: 0.2% to 11.3%)*
% obese	18.1%: 91/503	17%: 101/594	1.1% (95%CI: −3.4% to 5.7%)
% body fat >90^th^ percentile	F: 44.9% (131/292)M : 28.6% (58/203)	F : 30.8% (103/334)M : 22.4% (57/254)	14% (95%CI: 6.4% to 21.5%)*6.1% (95%CI: −1.9% to 1.4%)
% high blood pressure	20% (98/490)	11% (63/574)	9.0% (95%CI: 4.7% to 13.4%)*
% high cholesterol	15.4% (61/397)	11.7% (61/521)	3.7% (95%CI: −0.8% to 8.4%)
% high total cholesterol/HDL cholesterol ratio	8.7% (20/229)	2.9% (15/520)	5.9% (95%CI: 2.1% to10.4%)*
% high fasting glucose	6.1% (24/393)	1.2% (5/434)	5.0% (95%CI: 2.5% to 7.6%)*
% high fat in diet	71.1% (101/142)	52.3% (182/348)	18.8% (95%CI: 9.4% to 27.4%)*
% consuming 2 or more fruits per day.	17% (8/47)	47% (85/181)	30% (95% CI: 15% to 41%)*
Fitness (20 metre Multi stage fitness test average)	Boys: 48.49 (22.2)Girls: 31.9 (16.5)	Boys: 56.5 (21.0)Girls: 40.5 (13.1)	8.0 (95%CI: 3.4 to 12.5)8.6 (95%CI: 5.8 to 11.3)
% unfit	49.9% (168/337)	29.4% (160/544)	20.4% (95%CI: 13.8% to 26.9%)*
Median Fasting insulin	9.3 (IQR: 6.6–13.3)	7.9 (IQR: 5.3–10.6)	Mann-Whitney U test (p<0.001)
Median CRP	0.4 (IQR:0.2–1.1)	0.3 (IQR: 0.2–0.7)	Mann-Whitney U test (p<0.001)
Median adiponectin (IQR) µg/ml	4.216 (IQR:2.97–5.89)	2.83 (IQR:1.81–4.33)	Mann-Whitney U test (p<0.001)

* difference reaches statistical significance.

### Factors Associated with Obesity

**Figure 1:**
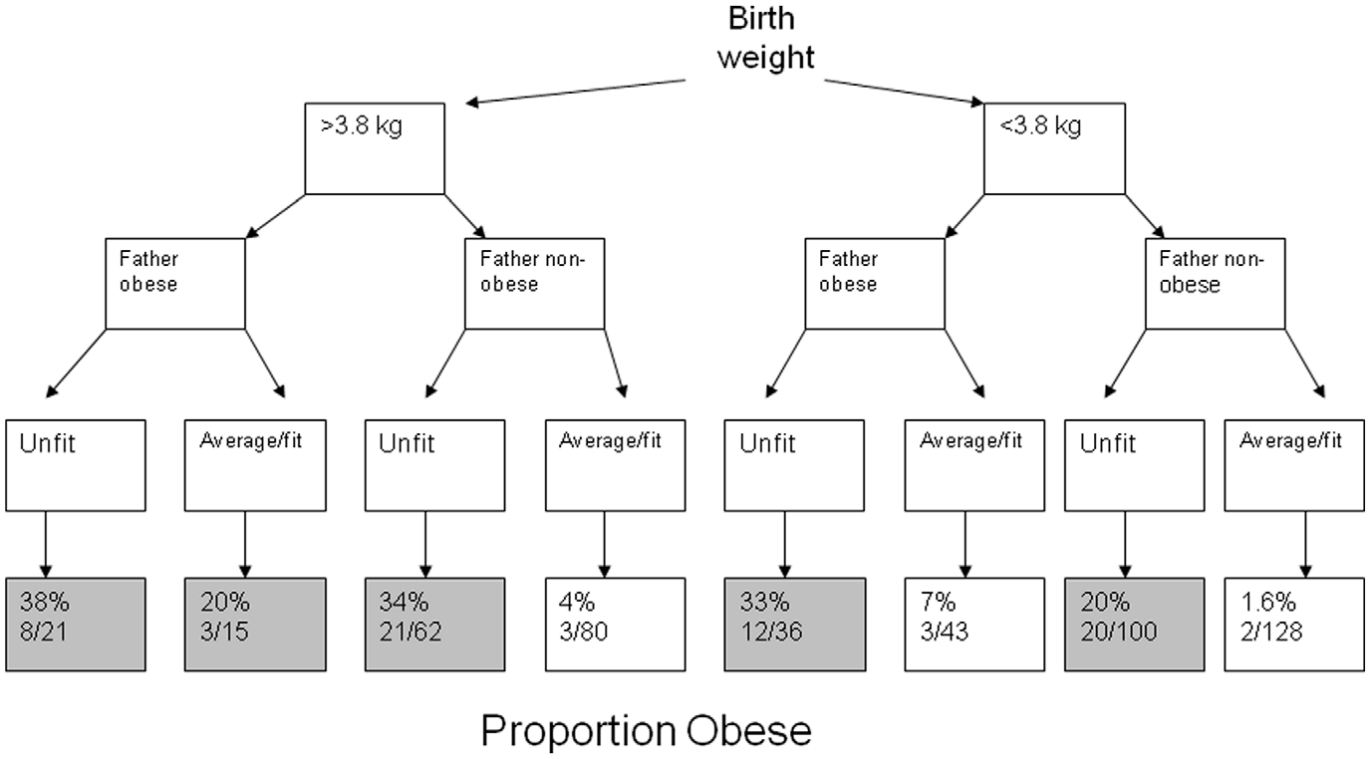
Obesity levels by risk factor.

Children classed as obese (Table 2), were more likely to have high blood pressure, high cholesterol, elevated fasting glucose, raised CRP and lower adiponectin, compared to non-obese children. Obese children were also more likely to have a higher birth weight, consume less fruit, have lower fitness score and have obese parents. When explanatory factors were combined in a mutually adjusted model, fathers’ BMI, birth weight and being unfit remained associated with obesity (Table 3 and Figure 1, Hosmer and Lemeshow statistic showed a good fit to the data (p = 0.6)). Factors no longer associated included mothers’ BMI and quantity of fruit consumed. Factors believed to be resulting from obesity were not included in the model (blood pressure, cholesterol, glucose CRP, adiponectin). Obese children were taller than non obese child but this factor was not included in the model as this was thought to be a result of increased calorie intake rather than a factor leading to obesity itself [8]. The mothers’ influence on daughters’ obesity, as well as fathers’ influence on sons’ obesity [9] was examined. In this dataset there was no evidence that mothers’ obesity influence daughters obesity. In fact, fathers’ obesity was more likely to influence daughters obesity. [See Table 4].

**Table 2:** Risk factors associated with obesity.

	Obese	Non-obese	Difference (95%CI)
Number (n = 1097)	192 (17.5%)	905 (82.5%)	
Female gender	60.9% (117/192)	57.1% (517/905)	3.8% (95%CI: −3.9% to 11.19%)
Ethnic minority group	19.0% (18/95)	12.3%(66/537)	6.7% (95%CI: −0.06% to 16.0%)
Birth weight Kg (standard deviation)	3.5 (0.6)	3.4 (0.6)	0.1 (95%CI: 0.004 to 0.2)*
Deprived school catchment area	47.4% (91/192)	45.5% (412/905)	1.9% (95%CI: −5.8% to 9.6%)
Free school meal eligibility (marker of deprivationfor individual child)	17.8% (28/157)	14.3% (109/763)	3.5% (95%CI: −2.3% to 10.6%)
Family history of high blood pressure	57.9% (55/95)	56.1% (303/540)	1.8% (95%CI: −9.1% to 12.1%)
Mothers BMI kg (standard deviation)Mother obese	27.7 (6.3)28.9% (31/107)	25.5 (5.0)16.0% (98/612)	2.2 (95%CI: 1.1 to 3.2)*13.0% (95%CI: 4.6% to 22.5%)*
Father BMI kg (standard deviation)Father obese	28.9 (4.9)36.8% (39/106)	27.2 (4.3)22.7% (121/532)	1.7 (95%CI: 0.7 to 2.5)*14.05% (95%CI: 4.7 to 24.1)*
TV in room	81% (64/79)	71.4% (309/433)	9.65% (95%CI: −1.1% to 18.0%)
Fibre in diet (gms per day)	11.8 (3.9)	12.4 (4.3)	0.63 (−0.33 to 1.6)
Number of fruit and veg per day	1.5 (1.3)	2.0 (1.9)	0.5 (95%CI: −0.09 to 1.2)
Fitness (20 metre Multi stage fitness test average)	Boys: 34.4 (15.7)Girls: 28.0 (11.5)	Boys: 57.2 (21.3)Girls: 39.1 (16.0)	22.8 (95%CI: 16.9 to 28.7)*11.1 (95%CI: 7.5 to 14.6)*
Height of child in metres	1.57 (0.08)	1.54 (0.87)	0.03 (95%CI: 0.02 to 0.04)*
High blood pressure	20.7% (38/183)	9.6% (83/868)	11.2% (95%CI: 5.5 to 17.9)*
% high cholesterol	19% (31/163)	11.9% (89/748)	7.1% (95%CI: 1.2% to 14.2%)*
Fasting glucose (mmol/l) (standard deviation)	4.95 (0.47)	4.9 (0.38)	0.06 (95%CI: 0.0 to 0.14%)*
Median Fasting insulin mU/l	13.95 (IQR: 9.9 to 19.2)	7.9 (IQR: 5.8 to 10.6)	Mann-Whitney U test*
Median Fasting CRP mg/l	0.9 (IQR: 0.4 to 1.8)	0.3 (IQR: 0.2 to 0.6)	Mann-Whitney U test*
Median Adiponectin µg/ml	2.95 (IQR: 1.81 to 4.36)	3.53 (IQR: 2.28–5.28)	Mann-Whitney U test*

* p<0.05.

**Table 3:** Adjusted model of factors associated with obesity.

Factors	Odds ratio	β Coefficient	P value
Unfit	8.8 (95%CI: 4.22 to 18.5)	2.18 (95%CI:1.44 to 2.9)	0.0001
Birth weight (kg)	1.6 (95%CI: 1.01 to 2.65)	0.49 (95%CI:0.01 to 0.97)	0.045
Fathers BMI (kg)	1.07 (95%CI: 1.01 to 1.13)	0.07 (95%CI:0.01 to 0.13)	0.021

**Table 4:** Obese levels stratified by gender.

*Both parents obese*			
	Daughter	Son	
Not obese	14 (66%)	7 (50%)	
Obese	7 **(33%)**	7 **(50%)**	
***Mother obese (father not)***			
Not obese	42 (84%)	19 (82%)	
Obese	8 **(16%)**	4 **(17%)**	
***Father obese (mother not)***			
Not obese	46 (75%)	48 (91%)	
Obese	15 **(25%)**	5 **(9.4%)**	p = 0.034*
***Both parents not obese***			
Not obese	197 (88%	142 (87%	
Obese	28 **(12%)**	22 **(13%)**	

* Daughters more likely to be obese compared to sons.

Mother and father BMI were combined using factor analysis to create a latent ‘family size factor’ and this variable was included in a mutually adjusted model excluding the variable of mother BMI and father BMI. Birth weight was no longer significant, and only fitness and ‘family size’ remained associated with obesity (odds ratio fitness: 9.66 (95%CI: 4.6 to 20), odds ratio family size: 2.33 (95%CI: 1.2 to 4.3)).

### Fitness and Obesity Combined (See Table 5)

**Table 5:** Risk associated with being obese and unfit.

Category	Healthy fitness andnon-obese N = 495	Healthy fitness andobese N = 43	Unfit and non-obese N = 215	Unfit and obese N = 96
High blood pressure	9%	13%	7.9%	22.9%*
High Cholesterol	9.79%	11.9%	14.9%	21.18%*
Median CRP (IQR)	0.3 (0.2–0.5)	0.5 (0.3–1)*	0.3 (0.2–1)	0.9 (0.4–1.7)*
Median insulin (IQR)	7.4 (5–9.8)	13.9 (10.7–21)*	8.35 (6.3–10.9)*	13.8 (9.8–19.2)*
Median glucose (IQR)	4.8 (4.6–5.1)	4.8 (4.5–5.0)	4.9 (4.7–5.2)	4.9 (4.7–5.1)

* significantly different compared to Healthy fitness and non-obese.

Of the 868 children measured, 57% (495) were non-obese and displayed average fitness in the 20 MST; 25% (215) were non-obese but unfit; 11.1% (96) were obese and unfit; while 5% (43) were obese but achieved average fitness scores on the 20 MST. The children who were obese and unfit were significantly more likely to have high blood pressure, cholesterol, CRP and fasting insulin levels. The children who were unfit (but not obese) were more likely to have higher fasting insulin compared to fit children, but they had comparable levels of other risk factors. The fit, but obese, children had higher levels of CRP and fasting insulin but did not have higher blood pressure or cholesterol compared to non obese children. It is possible that fitness may improve risk factors associated with heart disease (cholesterol and blood pressure), but not those associated with diabetes (insulin). Although the children with poor fitness and obesity had raised fasting insulin, this did not result in raised glucose levels at this age.

### Temporal Relationship Between Obesity and Fitness (See Figure 1)

Children who were large at birth (Figure 1) where more likely to be unfit (78%, 83/106) at age 11–13, compared to those who were not large at birth (44% (136/307, p = 0.001).

## Discussion

### Findings of This Study

A large proportion of children had at least one risk factor. For example, 1 in 3 children were overweight and of these, 60% were also unfit. A substantial proportion of children displayed factors predictive of future CVD and T2DM. These risk factors included, raised cholesterol, high blood pressure, high dietary fat intake and elevated fasting glucose. Children who were from a deprived area were at higher risk for all the risk factors (e.g. double the risk for high blood pressure). Children who were large at birth were at a higher risk of obesity especially if they were exposed to poor family behaviours (as seen in BMI of father). Combining mother and father BMI to give a ‘family size factor’ suggests that the influence of the mothers BMI is seen in the birth weight of the child as birth weight is no longer significant when mothers BMI is included in the model. Obesity is associated with risk factors for heart disease and diabetes. Maintaining fitness can reduce some of the risk factors for heart disease but has less effect on risk factors for diabetes (insulin levels). Both the school deprivation and the individual child deprivation levels were not associated with obesity. So, although risk factors of heart disease and diabetes were associated with deprivation, this association was not apparent for obesity.

The finding that childhood obesity is mainly associated with fitness, birth weight and fathers’ BMI strongly suggests that families need to be the main target for lifestyle interventions. The high levels of obesity among families with two obese parents (Table 4) provides a powerful argument for interventions such as MEND (http://www.mendprogramme.org/home) which works with families to address childhood obesity and improve fitness risk factors. However, the sheer number of ‘high risk’ pupils in each of our schools (between 31%–45% depending on the school), suggests that it may not feasible to target individual families, and that a societal and community approach is necessary. In terms of cost effective and sustainable strategies, it could be argued that public health interventions need to focus on the expectant mother and the fathers’ BMI if obesity rates are to be driven down. Our findings suggest that the problem needs to be addressed at the community and surrounding neighbourhood level rather than targeting the schoolchildren per se. Furthermore, we suggest that targeting fitness, particularly among girls with obese fathers, should be a priority.

### Strengths and Limitations

A major strength of the study is the objective measurement of the health of the child. However, some of our findings comparing deprived and non-deprived schools could have a bias due to the educational levels of the children in the different schools; notably, the diet diaries were completed in more detail by the non-deprived children. For example, the non- deprived children listed the vegetables included in the Sunday dinner, or in a vegetable curry, rather than simply writing ‘roast dinner with veg’, or writing ‘veg curry’. Ultimately, this allowed for the scoring and coding of the diaries to be more accurate for the non-deprived children. Although the same researcher conducted the 20 MST (shuttle test) in all schools, the teachers in some of the non-deprived schools were more actively involved in preparing the children for the test. Therefore, although we aimed to standardise the data collection across schools by having the same researcher in every school; the involvement or non-involvement of teachers in helping and advising participants might have introduced some bias in the results.

This study only examined collected data and did not impute missing data. The BMI measurements were only completed by 51% of fathers. It is possible that those fathers who completed questionnaires on their own BMI, were, in general, more actively involved in their child’s health; whereas the fathers who did not respond to the questionnaires had little influence on the child’s health.

### Findings in Context with Other Studies in the UK

The participants in this study appear to be less healthy than those participating in the SPEEDY study [10] in Norfolk, UK. Only 17% of the children were overweight in the SPEEDY study, compared to 29.5% of our non-deprived participants. However, the children in the SPEEDY study were, in general, from non-deprived schools (9% eligible for free school meals) which could explain some of the difference in physical activity and health. Our study supports the evidence of Sandercock et al [11] in suggesting that cardiorespiratory fitness is declining. This is further supported when comparing with our previous study [12] conducted 10 years ago. Using identical methods, we previously reported average boys’ and girls’ 20 MST at 57 (±21) shuttles and 40 (±14) shuttles respectively, compared to the present cohort’s 53.5 (±22.2) shuttles and 37.2 (±15.9) shuttles. We noted that adiponectin levels were higher in children from a deprived school (but not in overweight, or children with a high number of risk factors) compared to those from non deprived schools. This is surprising, and may be related to dietary habits or sleep patterns [13] To our knowledge, no previous publications have examined differences in adiponectin titre according to socio-economic level.

### Findings in Context with Other International Studies

The finding that fathers BMI is an important factor in child obesity strongly support findings by Freeman et al in an Australian population [14]. This study found an obese father with a healthy weight mother significantly increased the odds of child obesity; but the reverse scenario, namely, having an obese mother and normal weight father, was not associated with obesity. Our results would support this finding. In addition, Burke et al [15] found that fathers BMI predict child’s BMI independent of confounding variables and that BMI in children was consistently higher when fathers were overweight or obese, with obesity of fathers associated with a four-fold increase in risk of child obesity at age 18. A father’s, but not mother’s, total and percentage body fat have been found to be the best predictor of changes in daughters body fat [16]. This finding was supported by our results and highlights the importance of fathers’ BMI in child obesity. This may in part be due to fathers influence on eating behaviours [17] where fathers play a role in imposing child feeding practices and children of fathers with permissive and disengaged parenting styles had higher odds of being in a higher BMI category [18]. In addition, the father’s influence is important to a child’s involvement in exercise [19], with fathers more likely than mothers to have physically active play with the child [20]. For example, physical fitness of daughters are negatively related to fathers obesity [15], while fathers’ BMI is directly related to child’s activity level [21]. In education, there are many studies showing that the involvement of the father is important in educational achievement [22]–[24]; similarly, in mental health research fathers influence is critical [25]. Thus, fathers involvement is now recognised as important for many aspects of child health and development and this includes child obesity. In line with this, future intervention studies need to mindful of the importance of the father’s role.

The factors identified as associated with child obesity in this study were very similar to factors identified in other populations. For example, as with our study, in Australia [26] screen time did not predict obesity at age 14 (it did at age 8 and 10 years) but physical activity (using longitudinal data and structural equation modelling) did predict BMI in adolescence. In an American [27] study examining 6^th^ graders (age 11–12) 15% were reported as obese, which is similar to levels in our study. These children had higher cholesterol, blood pressure recovery heart rates, were less likely to engage in physical activity, ate school lunches and watched more TV. Thus, findings for risk factors associated with obesity were very similar across these populations. In Algeria [28], a school based study on children aged 6 to 8 years found 6.5% were obese and that obesity was associated mothers’ and grandmothers’ obesity, excess energy and fat intake, birth weight and low physical activity. Thus, although the obesity levels were lower, the factors associated with the condition were similar in this population. These findings suggest that targets to address childhood obesity are the same in most populations.

The Hands Study [26] and Metcalf study [29] found that fatness preceded inactivity with BMI at age 6 contributing to physical activity at age 8. We also found large birth weight (fatness) predicts low fitness at age 11–13. Therefore, there appears to be a spiralling pattern that being large at birth makes it harder to be active, being inactive leads to obesity, moreover, that obesity makes it harder again to be active. Thus, addressing factors which affect birth weight would help to address fitness in childhood, as well as obesity.

### Conclusions

Interventions to address childhood obesity should begin early. The expectant mother and father should be targeted with meaningful and practical lifestyle guidance. Our findings suggest that the promotion of fitness, particularly among children from obese families or who were large at birth, would reduce the likelihood of child obesity and of developing risk factors for future heart disease.

## Materials and Methods

The detailed methods of this cross sectional survey have previously been described in the protocol paper [30].

### Recruitment and Data Collection

The study population was recruited from ten schools. Schools were selected according to deprivation of the catchment area. All children in year 7 and 8 (i.e. aged 11 to 13 years) were eligible. These school years were chosen because they weren’t influenced by the pressures of external of examinations, the children were young enough to be amenable to lifestyle intervention changes and at the time of testing they had become accustomed to the school. Data collection occurred during the school year 2009/10. All testing procedures took place on school premises, and during allocated physical education lessons. The data collected are outlined in Table 1 and included; anthropometrical and physiological data, demographic data, physical activity, dietary intake, and blood samples for fasting lipids and glucose. In addition, children and parents completed a questionnaire detailing family history of diseases, parental BMI, birth weight and general health.

#### Anthropometrical and physiological data

The data collected included stature, body mass, skinfold thickness, neck, waist and hip circumference, blood pressure, and aerobic fitness. Participants wore shorts and t-shirt during anthropometrical assessment. Privacy was assured during all anthropometric measurements and two gender-specific adults were present at all times. Body weight was recorded to the nearest 0.1 kg using calibrated electronic weighing scales (Seca 770, Digital Scales, Seca Ltd, Birmingham, UK). Stature was measured using a portable stadiometer (Seca Stadiometer, Seca Ltd, Birmingham, UK). Body mass index (BMI) was calculated using the BMI formula of dividing the participants’ weight by their height squared [31]. BMIs were recorded, and participants were categorised as overweight or obese as defined by the International Obesity Task Force (IOTF) age and sex specific cut-offs for BMI, [32]. Biceps, triceps, subscapular and suprailiac skinfold measurements were taken on the right side of the body, using Harpenden skinfold callipers (John Bull, British Indicators Ltd, West Sussex, UK). Duplicate measurements were taken at each site and the average recorded. The sum of the four skinfold measurements were then calculated and recorded. Neck, waist and hip circumference was measured on each participant and recorded to the nearest millimetre using a standard flexible measuring tape (Rosscraft, UK). These provided an index of fat distribution [33] . High percentage body fat was taken as ≥56.9 mm for boys and ≥60.3 mm for girls [34]. systolic and diastolic blood pressure (BP) were measured using an Omron M6 automatic BP monitor (Omron Healthcare UK Ltd, Milton Keynes, UK) [35]. Blood-pressure was recorded three times, with the average of the second and third reading recorded for data analysis. Children were classified as having high blood pressure if the Systolic was >130 mm Hg or the diastolic was >85 mm Hg [36], [37]. Finally, aerobic fitness was measured using the 20 metre multi stage fitness test (20 MSFT), a valid and reliable method of assessing aerobic fitness in young people. Children were classed as unfit if they performed less than 49 shuttles for boys, and less than 27 shuttles for girls [34].

#### Measures of deprivation and ethnicity

The school was asked to complete a form providing information on the number of children receiving free school meals, thus identifying the deprivation level of the school [38]. Free school meals are allocated to pupils based on family income levels and are a marker of the individual child’s deprivation level. Schools were classified as being situated in a deprived catchment area if more that 21% of the school children were eligible to receive free school meals. This is an arbitrary cut off based on being above the Welsh average of 15% (95%CI: 14.8%–15.2%) of secondary school children being eligible for free school meals [39]. The deprived schools also scored highly on the Multiple Index of Deprivation [40], a measure based on levels of child poverty, unemployment, health deprivation and disability. The school records were used to identify the free school meal eligibility for each child and this was used as a marker for individual child deprivation level. Each child was also asked to self assign their ethnicity.

#### Physical activity and dietary intake

Physical activity was measured using the physical activity questionnaire for adolescents (PAQ-A). The PAQ-A is a seven day recall of physical activity; it is a valid and reliable measure of general physical activity levels that has been used widely in research [41]. The questionnaire uses memory cues such as lunch and evening items to enhance the recall ability of adolescents and provides a physical activity score derived from eight items, each scored on a 5-point scale.

Daily food intake was assessed using a validated, self reported seven day food diary [42]. Participants recorded everything they ate and drank over a specified seven-day period; during this time they were encouraged to continue with normal eating and drinking habits. Before keeping the food diary, a 45-minute oral training session was delivered to help participants describe intake and estimate portion size. The questionnaire included 12 semi-quantitative items to help clarify types of food and drink consumed. The food diary was analysed by Health Options Ltd (Health options Ltd, Cirencester, Gloucester, UK). Average daily kilojoules, percentage of total fat, saturated fat, carbohydrate, protein, fibre and number of fruits and vegetables consumed on average per day were calculated. Children were categorised as having high fat consumption if more than 33% of their total calories came from fat [43].

#### Lipids and lipoproteins

Venous blood samples were taken from each consenting participant. These were taken first thing in the morning whilst the children were in a fasted state. The children were required to report any recent episodes of acute illnesses or infections, and whether they were regular smokers as these conditions could cause elevated CRP concentration. To control for plasma volume shifts, venous blood was sampled after the children had assumed a seated position for at least 30 minutes. Blood samples were taken by qualified phlebotomists, with a nurse or doctor present at all times. Blood samples were analysed for fasting levels of glucose, insulin, lipids, high molecular weight adiponectin and high sensitivity C-reactive protein (CRP). Age and gender specific cut off points proposed by the International Diabetes Federation (IDF) [37] were used for the biochemical risk factors of high triglyceride (≥1.7 mmol/L), low levels of high density lipoprotein cholesterol (<1.03 mmol/L), total cholesterol/High density lipoprotein ratio of >4, and elevated blood glucose (≥5.6 mmol/L) [37], [44].

Parents and children were asked to complete a questionnaire regarding family history of conditions, parents height and weight, ethnic background. However, this questionnaire was only introduced after the first 4 schools had been recruited and participated. Therefore, not all children and parents were given this background questionnaire.

Ethics approval for this study was granted by the Local NHS Research Ethics Committee- Dyfed Powys REC. Written parent and child consent was gained for each participant.

#### Statistical analysis

STATA release 8 was used for all analysis. Factors were tested for normality and if skewed the medians or proportions were presented. Descriptive analysis was used to explore factors associated with obesity and factors associated with being unfit were similarly investigated. Logistic regression was performed using step down selection. All factors associated with high risk from the initial unadjusted analysis were entered into the model and likelihood ratio tests was used to reduce the adjusted model. School was included as a random effect within the model to adjust for clustering by school. Goodness of fit was assessed using the Hosmer and Lemeshow statistic [45]. Factor analysis was used to combine mother and father BMI to create a latent ‘family size’ variable for inclusion in the logistic regression analysis. Only data collected were analysed, no imputations or estimates of missing data variables were conducted.
