# Correction: Child Fitness and Father’s BMI Are Important Factors in Childhood Obesity: A School Based Cross-Sectional Study

**DOI:** 10.1371/annotation/62537085-2630-490b-b489-8dfdcc3a84fa

**Published:** 2012-08-29

**Authors:** Sinead Brophy, Anwen Rees, Gareth Knox, Julien S. Baker, Non E. Thomas

There was an error in the fourth author's name. Julien S. Baker is correct. The correct citation is: Brophy S, Rees A, Knox G, Baker JS, Thomas NE (2012) Child Fitness and Father’s BMI Are Important Factors in Childhood Obesity: A School Based Cross-Sectional Study. PLoS ONE 7(5): e36597. doi:10.1371/journal.pone.0036597. Correct author contributions for Julien S. Baker (JSB) are: Designed the study, Was involved in the conception, design, and/or acquisition of the data, Supervised all aspects of the work and revised drafts, Gave final approval. 

